# Canine Distemper Virus: Origins, Mutations, Diagnosis, and Epidemiology in Mexico

**DOI:** 10.3390/life14081002

**Published:** 2024-08-13

**Authors:** Alejandra Rivera-Martínez, Carlos A. Rodríguez-Alarcón, Jaime R. Adame-Gallegos, S. Viridiana Laredo-Tiscareño, Erick de Jesús de Luna-Santillana, Luis M. Hernández-Triana, Javier A. Garza-Hernández

**Affiliations:** 1 Instituto de Ciencias Biomédicas, Universidad Autónoma de Ciudad Juárez, Juárez 32310, Chihuahua, Mexico; alejandra.riveramtz0@gmail.com (A.R.-M.); carrodri@uacj.mx (C.A.R.-A.); viridiana.laredo@gmail.com (S.V.L.-T.); 2 Facultad de Ciencias Químicas, Universidad Autónoma de Chihuahua, Chihuahua 31125, Chihuahua, Mexico; jadame@uach.mx; 3 Laboratorio Medicina de la Conservación, Centro de Biotecnología Genómica del Instituto Politécnico Nacional, Reynosa 88710, Tamaulipas, Mexico; edeluna@ipn.mx; 4 Animal and Plant Health Agency, Virology Department, Vector Borne Diseases Research Group, Addlestone KT15 3NB, UK; luis.hernandez-triana@apha.gov.uk

**Keywords:** canine distemper virus, origins, mutations, diagnosis, epidemiology, PCR, Mexico

## Abstract

This review provides an overview of the canine distemper virus (CDV), a highly infectious pathogen causing severe disease in domestic dogs and wildlife. It shares genetic similarities with the human measles virus (HMV) in humans and the rinderpest virus (RPV) in cattle. The origin of CDV likely involves a mutation from human measles strains, possibly in the New World, with subsequent transmission to dogs. CDV has been globally observed, with an increasing incidence in various animal populations. Genomic mutations, especially in the H protein, contribute to its ability to infect different hosts. Diagnosis by molecular techniques like RT-qPCR offers rapid and sensitive detection when compared with serological tests. Genomic sequencing is vital for understanding CDV evolution and designing effective control strategies. Overall, CDV poses a significant threat, and genomic sequencing enhances our ability to manage and prevent its spread. Here, the epidemiology of CDV principally in Mexico is reviewed.

## 1. Introduction

**Figure 1:**
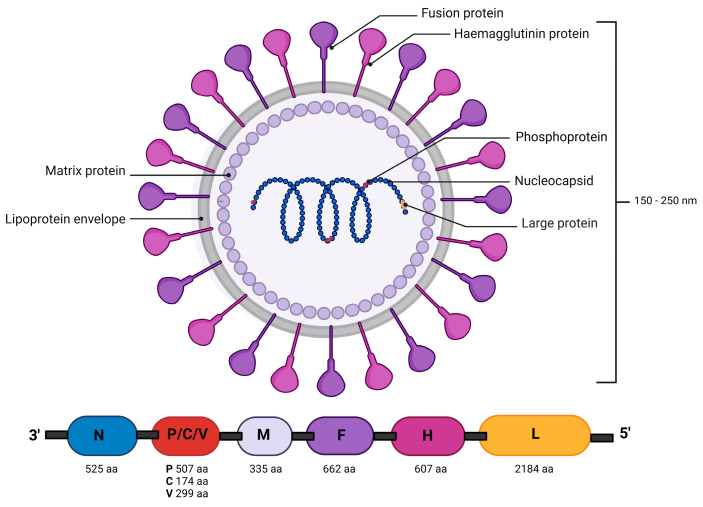
Morphology and organization of the genome and proteins of the CDV virion particle. The codified proteins of CDV are N: nucleocapsid, P: phosphoprotein, M: matrix protein, F: fusion protein, H: hemagglutinin, L: large polymerase protein; P: a gene that encodes the C and V proteins. Image created in Biorender (https://www.biorender.com).

Canine distemper virus (CDV) is responsible for a multisystemic disease commonly known worldwide as canine distemper or distemper, also referred to as Carré’s disease. The first scientific report on this disease was made by Antonio de Ulloa in 1735 in Ecuador and Peru. He observed and described findings of encephalitis that produced neurological signs, but without the aggression reported in dogs with rabies, and with the absence of transmission through bites [1,2]. Henri Joseph Carré in 1905 was the first to identify a filterable virus in the serous nasal discharges of dogs affected by canine distemper. Furthermore, it was observed that after a filtration process, two or three drops could induce the disease and cause death in susceptible dogs [3,4]. Subsequently, confirmation occurred in 1926, by Dunkin and Laidlaw. CDV presents variable forms, usually manifesting as spheres with a diameter ranging from 150 to 250 nm [5]. Its genome is a single-stranded non-segmented negative-sense RNA, composed of 15,616 nucleotides encoding eight proteins, of which six are structural and two are non-structural [6] (Figure 1). Belonging to the Morbillivirus genus within the Paramyxoviridae family, CDV shares genetic connections with other viruses capable of infecting both humans and animals [7]. These Morbilliviruses that correlate human–animal diseases represent some of the most devastating “collective” diseases: human measles virus (HMV), rinderpest virus (RPV) in cattle (eradicated in 2011), and CDV. These viruses share similar characteristics, such as their ability to be transmitted directly and their high rates of morbidity and mortality in populations without previous exposure, positioning them among the most infectious known viruses [8]. Now, zoonoses are typically known for being transmitted from animals to humans. However, CDV is unique because has mutated from initially affecting humans (measles) to now affecting dogs (distemper), leading to severe, potentially deadly meningitis [7,8]. Although there are no documented cases of CDV infecting humans, some researchers have linked the virus to Paget’s disease, a bone disorder in humans [7]. This association has led to the suggestion that CDV could potentially become a zoonotic threat, meaning it might be transmitted from animals to humans under certain conditions. This review aims to compile crucial information on the origins and mutations of CDV, detail diagnostic methods used by public health personnel, and present important epidemiological data on CDV in Mexico. Also, it holds significant value for the scientific community, particularly in developing countries with environments similar to Mexico, where large populations of stray dogs often lack adequate veterinary care. The challenge of preventing CDV is heightened in these regions, as stray dogs can serve as reservoirs for the virus, posing a threat not only to household dogs, including those that are vaccinated, but also to susceptible wildlife. The findings and conclusions of this review could provide crucial insights and strategies for controlling the disease in similar international contexts.

## 2. Materials and Methods

**Figure 2:**
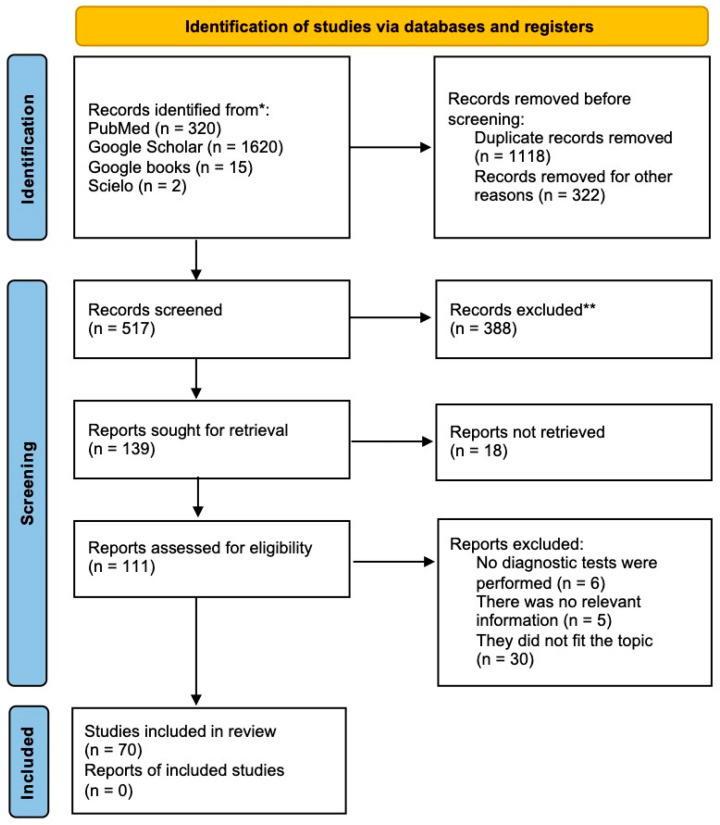
PRISMA analysis. Selection process of scientific literature on CDV, its origin, genomic mutations, serology and diagnosis, and epidemiology in Mexico using the PRISMA algorithm (Preferred Reporting Items for Systematic Reviews and Meta-Analyses). * Number of records identified from each database or register searched. ** Records excluded by a human; no automation tool was used.

To compile this review, we followed the protocol of the Preferred Reporting Items for Systematic Reviews and Meta-Analyses (PRISMA) checklist (see Supplementary File), accurately following its steps for identifying, analyzing, selecting, and incorporating pertinent literature [9] (Figure 2). The search was carried out using academic search engines and databases including PubMed, Google Scholar, Google Books, and SciELO (Scientific Electronic Library Online). The search criteria used were in both English and Spanish, utilizing a careful set of keywords including “canine distemper”, “genotypification”, “PCR”, “zoonosis”, “México”, “animal”, “detection”, “virus”, epidemiology”, and “diagnosis”. The search parameters were not restricted by publication year, encompassing literature dating back to the 20th century and extending up to December 2023.

## 3. Results Overview: Insights into Canine Distemper Virus

### 3.1. Hypothesis on CDV’s Origin

The human measles virus (MV), which affects both humans and non-human primates, exhibits a close affinity with CDV; besides belonging to the *Paramyxoviridae* family, they share the characteristic of being enveloped viruses with single-stranded negative-sense RNA and non-segmented genomes [10]. Notable similarities in nucleotide and amino acid sequences between these two viruses result in significant levels of functional and structural conservation. Therefore, CDV has been used as an effective model to understand the measles pathogenesis [11]. Measles infection is generally self-limiting in humans, and the occurrence of central neurological signs is rare. However, CDV infection in dogs leads to a more severe pathology. Affected dogs often develop complications in the central nervous system, and the mortality rate, depending on the strain, can be very high, varying at between 50 and 90% [12,13,14]. It is essential to highlight that, although CDV generates a similar pathogenesis in different hosts, the severity of the disease varies, and it can be entirely lethal in highly susceptible species, such as ferrets and various wild carnivores [15]. Clinical signs in the acute phase of both infections are comparable and include fever, a distinctive skin rash, diarrhea, nasal discharge, conjunctivitis, and generalized immunosuppression. These symptoms reproduce the clinical spectrum observed in patients affected by the measles virus [16]. Researchers investigating canine distemper in the 19th and early 20th centuries supported the hypothesis that CDV originated through humans, based on the similarities between both diseases. In summary, the existence of a zoonosis was proposed in which humans transmitted the infection to dogs. In fact, in the early stages, the treatment for dogs infected with CDV involved the injection of human blood, as antibodies generated by measles could protect against CDV. Additionally, studies by Uhl et al. [8] support the idea that CDV emerged in the New World. This is based on their analysis of contemporary virus sequence data, showing the adaptation of European measles strains to dogs. Factors such as genetic interchangeability, the number of amino acids, nucleotide and amino acid sequences, as well as antigenic epitopes present in HMV, CDV, and RPV Morbilliviruses demonstrate a high degree of conservation. According to phylogenetic reconstructions, HMV, CDV, and RPV Morbilliviruses are suggested to share a unique common ancestor [17]. This is demonstrated in the Bayesian phylogenetic reconstruction, where the Morbillivirus group is clearly defined and shares a common ancestor denominated as morbilli-related viruses (UMRVs) [18].

**Figure 3:**
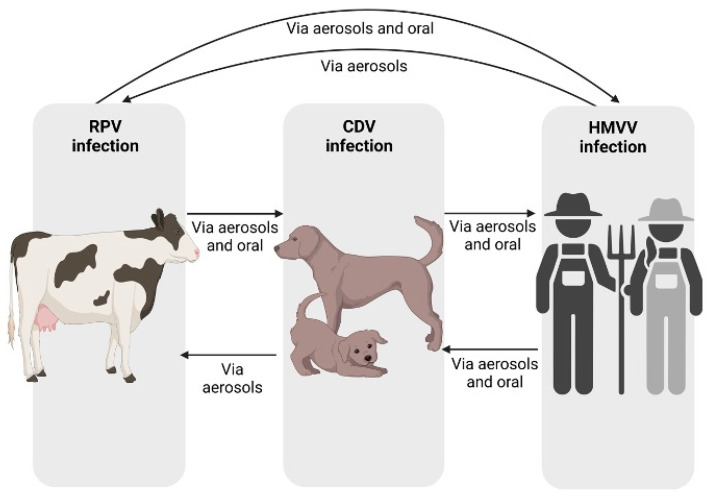
CDV infection cycle. Different modes of transmission of RPV, HMV, and CDV viruses between species. Image created in Biorender (https://www.biorender.com).

These three Morbilliviruses are notable for their ability to transmit directly (Figure 3) and the characteristic of using the same cellular receptors.

Current diagnostic methods involve serological, molecular, transmission electron microscopy (TEM), and sequencing tests, while in the past, they were primarily differentiated based on the infected host. Numerous Morbillivirus epidemics have been described in both humans and animals. Among them are measles (around 900 AD) and rinderpest (around 376 BC), first reported in regions of Europe, Asia, and the Middle East. In contrast, the first description of CDV was recorded much later, specifically in 1748 AD, in South America, by the scientist Antonio de Ulloa. For this reason, it has been established that HMV reached the Americas in the 15th century with European colonizers, mutated, and caused the first cases of CDV. After the initial report of CDV, various epidemics occurred in Europe. It has been described that the virus reached Europe in 1760, with its specific introduction in Spain. A notable event was the 1763 epidemic in Madrid, during which 900 dogs died in a single day. In 1764, the disease was diagnosed in Great Britain and Italy, and in Russia in 1770. In summary, there is a possibility that CDV could have originated in South America through a mutation of HMV and infected dogs, who then brought it to Europe [1,2,19]. It is important to note the lack of paleopathological and historical evidence supporting the presence of CDV in the New World before the arrival of Europeans. A thorough analysis of 2335 dog teeth from a collection of pre-Columbian skeletons in America revealed no characteristic lesions of CDV infection, suggesting that the virus was not present in the New World before the arrival of Europeans [12].

### 3.2. Epidemiology of CDV in Mexico

Since the 1980s, an increase in the incidence of CDV has been observed in various regions such as the United Kingdom, Australia, Switzerland, and New Zealand, accompanied by a rise in vaccine failures [20]. Although initially described as the causal agent of an infectious disease in domestic dogs, CDV is now recognized more as a pathogen affecting a wide variety of carnivores. Numerous outbreaks of CDV have been recorded in various wildlife species, highlighting the lack of knowledge about CDV susceptibility in these emerging contexts [6]. Epizootics caused by CDV in wild animals are a serious global issue. For instance, the discovery in 1994 of a large number of dead lions in the Serengeti National Park in Tanzania was attributed to a CDV infection. In California, the island fox population has declined due to CDV epidemics. Cases of CDV have been observed in various wild animals from different regions in Japan. Additionally, CDV infections have been documented in rhesus monkeys in China, and they were noted in macaques in Japan in 2008 [21,22]. CDV infections have been detected in all terrestrial carnivore families, including Canidae, Felidae, Mustelidae, Ursidae, Viverridae, Hyaenidae, and Procyonidae [23].

CDV is a serious disease for carnivores and other mammals, with limited studies in wildlife. In 1999, the population of island foxes (*Urocyon littoralis catalinae*) on Santa Catalina Island, California, decreased by approximately 95% in their eastern range. This reduction was attributed to a co-infection of canine distemper virus (CDV) and toxoplasmosis. It was found that the CDV was related to that of continental raccoons. Serological evidence indicated a higher exposure to CDV in foxes during 1999–2000 compared to 1998, suggesting that CDV was the main cause of the decline [24]. Rodríguez-Cabo-Mercado et al. investigated a CDV outbreak in a community of common raccoons and white-nosed coatis, finding an index case in a raccoon. Techniques such as seroneutralization, RT-PCR, and immunofluorescence were used for diagnosis and analysis. The prevalence of CDV was 19.6% in coatis and 25.3% in raccoons, with significant differences in the immune response between the two species. A new CDV sequence was identified, similar to Asian and European lineages, and an endemic state of CDV was observed with different dynamics between the studied species [25].

Although Gamiz et al. [26] argue that canine distemper is one of the main viral diseases affecting dogs in Mexico, the number of epidemiological studies in the country is insufficient. Few investigations have been carried out by university institutes in various regions such as Jalisco, Estado de México, Nuevo León, and Coahuila. Some notable studies include González-Vallejo’s [27] research in Monterrey, which, using molecular techniques, reported a 71% prevalence of CDV in dogs with a canine respiratory complex. Additionally, García-Vidaña [28] found a prevalence of 23.3% in a small population in Coahuila. In 2018, a retrospective study at the Autonomous University of Baja California revealed an overall prevalence of 1.8% of sick dogs between 2015 and 2017. In Mexicali, a substantial increase in CDV prevalence was identified between 2015 (0.2%) and 2016 (3.8%), with a 19-fold higher probability of diagnosis in the latter year. In 2017, veterinary clinics reported an increase in patients diagnosed with canine distemper [29]. Finally, Rebollar-Zamorano et al. [14] conducted an epidemiological study in the state of Hidalgo, determining a prevalence rate of 9 cases per 1000 sick dogs treated at the investigated veterinary hospital. In Chihuahua, a study was conducted in an urban transition area in Janos, revealing that a total of 62% of domestic dogs showed antibodies against CDV, especially in free-roaming domestic dogs; they also found seropositivity in a red lynx (*Lynx rufus*) [30].

### 3.3. Genomic Mutations of CDV

According to Zhao et al. [15], the nature of the CDV genome leads to high mutation rates, generating extensive genetic diversity in this virus. The H protein plays a crucial role in determining cellular tropism and the host range by interacting with the SLAM receptor present in lymphoid tissues [31]. It is noteworthy that the H gene exhibits the greatest variability in the Morbillivirus genome. In its amino acid sequence, divergence values of 8% are observed between field isolates and up to 11% compared to vaccine strains [32]. The high genetic heterogeneity of hemagglutinin has allowed for studies of genetic and phylogenetic analysis, including the description of the global distribution of CDV genotypes. This has led to the classification of surrounding strains into 17 different lineages [6]. It is important to highlight that some mutations in this protein are responsible for allowing the virus to expand its infection range, facilitating its ability to infect various hosts. The molecular analysis of CDV strains has suggested that amino acid residues 530 and 549 in the hemagglutinin (H) protein are associated with host specificity [33]. Substitutions at these two key positions are related to cross-species transmission, based on the specificity of glycosylated H protein, which is involved in receptor binding and is much more variable than other CDV proteins. This makes it an appropriate gene for investigating the genetic diversity of the virus [34]. In previous studies, it was found that when the canine strain of CDV was passed in ferrets (Mustela putorius), the H protein acquired a mutation leading to the substitution of tyrosine with histidine at amino acid position 549. CDV with histidine at residue 549 was highly virulent for raccoons compared to strains lacking this substitution [33]. Sequence analysis of the hemagglutinin gene and comparison with wild-type CDV from different species in the same geographical areas identified two non-synonymous single-nucleotide polymorphisms in 10 CDV strains, leading to amino acid changes at positions 542 (isoleucine to asparagine) and 549 (tyrosine to histidine) of the H protein coding sequence. The change at residue 542 generated a potential new glycosylation site. This masking of antigenic epitopes by sugar residues could represent a mechanism for evading neutralizing antibodies and reduced protection through vaccination [33].

### 3.4. Advances in the Diagnosis of CDV

The clinical diagnosis of CDV often poses challenges for veterinarians due to the wide range of clinical signs, due to their similarity to those caused by other viral agents such as canine parvovirus, canine parainfluenza virus, canine adenovirus type 2, or canine coronavirus [35]. For this reason, various diagnostic methodologies have been developed, including serological and molecular techniques. Clinical diagnosis relies on the history and clinical signs of animals, which generally indicate multisystem involvement. The virus has been found persistently in different tissues, such as the external lining of affected areas like footpads, urothelium, and the uvea.

One challenge in diagnosis is that signs and symptoms of the disease are not very evident in the early stages, especially in wildlife. Consequently, accurate and rapid diagnosis is essential for effective disease management and control [36]. Virus isolation is the gold standard for diagnosing viral diseases. However, with CDV, there is a reported low success rate in viral isolation due to its high sensitivity to light and temperature. Additionally, virus titration, specific cell lines, and dedicated facilities are often required [37,38]. Serological antibody detection tests are considered reference tests as they quantitatively reveal the types of antibodies present [39]. It is important to note that dogs develop lifelong protection against canine distemper virus by producing high levels of neutralizing antibodies (1:100) after infection, peaking in 2 to 3 weeks. An antibody titer of 1:32 is considered a threshold indicator of protection against CDV infection, although some studies have found protective titers in the range of 1:80 to 1:160. Lastly, a more than fourfold increase in IgG titer within 14 days suggests infection, even in recently vaccinated animals [36,40]. The gold standard for these tests is viral neutralization (VNT), but it requires a considerable amount of time and conventional virology skills, as well as handling live viruses. This makes other immunoassays like the enzyme-linked immunosorbent assay (ELISA) and immunofluorescence (IF) preferable [41]. ELISA tests rely on the detection of antibodies using monoclonal antibodies and chromatographic analysis. Qualitative samples of antibodies can be obtained from various secretions, such as conjunctival, nasal, saliva, urine, serum, or plasma [42]. ELISA is widely used to detect IgM and IgG antibodies up to three months after infection in both dogs and other hosts. This test provides specificity and sensitivity as consistent as the VNT [36]. For example, one study reported that the accuracy of CDV ELISA tests equates to a 94.0% sensitivity and 91.8% specificity [43]. Immunofluorescence is based on demonstrating antigens in conjunctival, vaginal, and nasal swabs, tracheobronchial washes, and urine sediment using polyclonal or monoclonal antibodies [32,44]. Although external mucosae offer a straightforward and highly cellular sampling, the sensitivity of the test at this site is around 80%.

On the other hand, immunohistochemistry is a technique based on staining fixed tissues embedded in paraffin. These assays are used to detect inclusion antigens or antibodies in bronchial cells, lymph nodes, the urinary bladder, or nervous system tissues. It is essential to note that they provide reliable results only when marked viremia is recorded [45]. Molecular assays offer sensitive and specific detection in both ante- and post-mortem samples.

Unlike serological tests, these assays are based on sequencing and amplifying CDV nucleic acids for diagnosis. These techniques amplify genomic regions conserved among phylogenetically close viruses to CDV, ensuring the reliability of the results. The reverse transcription combined with polymerase chain reaction (RT-PCR) molecular technique provides highly accurate results by identifying the presence of viral RNA in infected cells. This technique involves the reverse transcription of RNA to generate complementary DNA, allowing subsequent amplification of specific fragments using primers targeted at genes used for sequencing (Table 1). RT-PCR stands out for its sensitivity, specificity, and speed, making it an effective option for early virus detection [46,47]. It is most specific and sensitive in detecting CDV in whole blood, serum, and cerebrospinal fluid [36,48]. In contrast, nested PCR shows superior sensitivity in diagnosing CDV in clinical samples of urine, blood, and saliva compared to RT-PCR and immunofluorescence assays [49]. Finally, real-time reverse transcription polymerase chain reaction (RT-qPCR) using a TaqMan probe based on CDV-N and P genes is highly sensitive and specific compared to other tests [36]. Sequencing genomic fragments of CDV after amplification by PCR is essential to provide detailed information about its genetic composition. This allows for precise identification and complete characterization of the virus, including revealing specific nucleotide sequences and genetic variations present. Another sequence-based genomic technique is next-generation sequencing (NGS), which is also called metagenomics. This is a cutting-edge technology that enables the comprehensive sequencing of genomes in a sample. Important viral metagenomics studies have been performed on wild and domestic canids, increased our understanding of the viromes [50,51,52]. Recently, NGS proved to be a significant tool for the rapid genomic characterization of CDV strains during a likely widespread epizootic event among foxes in Hungary in 2021. NGS demonstrated its ability to obtain 19 complete genomes of CDV strains quickly and with high precision, showcasing its great potential for monitoring pathogens in wildlife, including CDV [53].

These molecular approaches are crucial not only for epidemiological and phylogenetic studies but also for monitoring the virus’s evolution, identifying relevant mutations, and understanding the genetic relationships between different strains. The information obtained contributes to our understanding of the genetic diversity of CDV and classifying lineages, supporting the design of more effective control and prevention strategies, as well as the development of vaccines adapted to circulating virus genetic variants. In summary, genomic sequencing is an essential tool to advance the management and understanding of CDV infection. Table 1 shows the main diagnostic methods used for the detection of CDV, as well as the detection target and some important notes on the diagnostic test.

Another important technique with a significant impact on veterinary pathology is transmission electron microscopy (TEM). TEM is a high-resolution imaging method essential for confirming morphological similarities within a group due to its ability to provide detailed images of cellular and molecular structures at the nanometer scale [54]. In the case of CDV, TEM has been widely used to understand the disease’s pathology. For example, it has been employed to visualize virus particles and cellular damage in captive raccoons (Procyon lotor) [55], and to investigate the cell tropism of CDV in an ex vivo model of pulmonary infection in dogs [56].

**Table 1:** Common diagnostic methods used for the detection of CDV.

Diagnostic Method	Detection Method	Type of Diagnostic Method	Target	Notes on the Test	Reference
MDCK, MV1 Lu, cells Vero-SLAM B95a	Virus isolation	Cell culture	Virus	Gold-standard test, but currently infrequently used	[57]
Direct ELISA	Antigen detection	Serological	CDV antigen	Detects antigen in serum	[58]
Sandwich ELISA	Antigen detection	Serological	Protein H antigen	High specificity Detection and quantification	[59]
			Protein F antigen	Efficient in field application with fecal and serum samples	[60]
Sandwich dot ELISA	Antigen detection	Serological	Virus	Rapid test, used in epidemiological surveillance	[61]
LFA	Antigen detection	Serological	Protein F antigen	Rapid test	[62]
Immunofluorescence	Detection of fluorescently labeled antibodies	Immunofluorescence	Protein F/H antigen	Laborious test, a fluorescence microscope is needed, highly sensitive and specific	[40]
RT-PCR	RNA detection	Genomic	Gen N	Standard laboratory test, currently the most used	[63]
One-step nested RT-PCR	Antigen detection	Genomic	Gen N	100 times greater sensitivity than RT-PCR and nested PCR	[38]
Double-step real-time RT-PCR	RNA detection	Genomic	Gen N	Highly sensitive and specific Quantify the viral load in clinical samples	[7]
One-step real-time RT-PCR	RNA detection	Genomic	Gen N	Allows the study of viral replication and the kinetics of the viral load of viral RNA in infection	[64]
RT-LAMP assay	RNA detection	Genomic	Gen H	100 times more sensitive than RT-PCR Only 1 h of reaction	[65]
RT-qPCR	RNA detection	Genomic	Protein M gene and MF intergenic region	Uses the TaqMan probe based on the CDV-N and P genes and is highly sensitive and specific compared to other tests	[66]
RT-PCR-RFLP	RNA detection plus restriction enzymes	Genomic	N gene amplification plus BamHI Restriction enzyme (RE) digestion Gen N amplification plus enzyme digestion using MspI	It results in a different number of fragments in both strains	[67]
ELISA	Detection of specific antibodies against the virus	Serological	Antibody IgG	Detect within 6 days of infection	[68]
Dot blot assay	Detection of specific antibodies against the virus	Serological	N protein-specific IgM	Detect recent infections	[69]
Capture sandwich ELISA	Detection of specific antibodies against the virus	Serological	N protein-specific IgG and IgM antibody	No cross-reactivity with other morbilliviruses	[70]

Abbreviations: BamHI: *Bacillus amyloliquefaciens* HI restriction enzyme. IgG: Immunoglobulin G type. IgM: Immunoglobulin M type. LFA: Lateral flow assays. MDCK: Madin–Darby canine kidney. MspI: Isoschizomer of HpaII restriction enzyme. MV1 Lu: Mink lung epithelial cell line. qPCR: Quantitative polymerase chain reaction. RT-LAMP: Reverse transcription loop-mediated isothermal amplification. RT-PCR-RFLP: Reverse transcription polymerase chain reaction restriction fragment length polymorphism. RT-PCR: Reverse transcription polymerase chain reaction. Vero-SLAM: Cells from kidney of an African green monkey encoding the human signaling lymphocytic activation molecule (SLAM).

## 4. Conclusions and Future Perspectives

CDV causes a multisystemic disease in domestic dogs and wild carnivores, with high morbidity and mortality. This virus shares genetic similarities with the human measles virus (HMV). Historical hypotheses suggest a mutation from European strains of human measles to dogs in the New World. Globally, there is an observed increase in CDV incidence in both dogs and wildlife. Genomic mutations of CDV, especially in the H protein, generate genetic diversity, influencing its ability to infect various hosts. Diagnosis poses a challenge for veterinarians due to difficulties in detecting antibodies through serological tests and identifying the virus through molecular methods. However, RT-qPCR stands out for its sensitivity and speed in viral RNA detection. Finally, genomic sequencing plays a crucial role in understanding the evolution of CDV and developing effective control strategies. This technique provides crucial details that help us to understand the genetic diversity of CDV and enhance infection management. Thanks to the information obtained through genomic sequencing, more precise and tailored approaches can be developed to prevent and control the spread of this disease.

## Data Availability

Not applicable.

## Supplementary Materials

The following supporting information can be downloaded at https://www.mdpi.com/article/10.3390/life14081002/s1, PRISMA checklist.
